# A Sulfated Abalone Polysaccharide Inhibited SARS-CoV-2 Infection of Vero E6 Cells In Vitro

**DOI:** 10.3390/foods11182865

**Published:** 2022-09-16

**Authors:** Jinghe Sun, Shuang Song, Chunqing Ai, Beiwei Zhu, Jingfeng Yang

**Affiliations:** Collaborative Innovation Center of Seafood Deep Processing, National Engineering Research Center of Seafood, School of Food Science and Technology, Dalian Polytechnic University, Dalian 116034, China

**Keywords:** sulfated, polysaccharide, SARS-CoV-2, biolayer interferometry, ACE2

## Abstract

Sulfate polysaccharides, such as heparin sulfate, have been found to have inhibitory activity against SARS-CoV-2. An abalone polysaccharide, AGSP, was deeply sulfate modified using the chlorosulfonic acid/pyridine method, yielding S-AGSP. AGSP and S-AGSP inhibitions of SARS-CoV-2 infection of Vero E6 cells were tested in vitro. The interference of AGSP or S-AGSP on the binding interaction between the SARS-CoV-2 spike protein and angiotensin-converting enzyme was tested using a biolayer interferometry assay. Results showed that S-AGSP, above a concentration of 1.87 µg/mL, significantly inhibited SARS-CoV-2 infection of Vero E6 cells. Compared with AGSP, S-AGSP obviously weakened the affinity between the SARS-CoV-2 spike protein and ACE2. The polysaccharide’s sulfate content played a vital role in influencing the binding affinity of spike protein to ACE2. Therefore, S-AGSP has potential as a COVID-19 competitive inhibitor as well as a candidate to be repurposed as a prophylactic COVID-19 therapeutic.

## 1. Introduction

COVID-19 is a new infectious disease that quickly spread across the world early in 2020. This disease is caused by the severe acute respiratory syndrome virus associated with coronavirus-2 (SARS-CoV-2) and has become a primary public health threat [1]. To date, some research regarding SARS-CoV-2 pathogenesis has been carried out to help understand this virus’ invasion process of a host cell. Upon initially contacting the surface of a host cell, SARS-CoV-2 binds to cellular receptors using the spike glycoprotein (comprising an S1 subunit and an S2 subunit in each spike monomer) on the envelope. SARS-CoV-2 utilizes the glycosylated spike protein to bind to a host receptor’s angiotensin-converting enzyme 2 (ACE2); this is a critical initial step facilitating the virus’ entry into target cells [2,3]. This binding triggers a cascade of events, which lead to the fusion of the cell and viral membranes allowing the virus to enter a host cell [4].

Recent studies reported that soluble unfractionated heparin and its derivatives inhibited SARS-CoV-2 host cell entry in vitro [5]. A sulfated polysaccharide isolated from sea cucumber was also identified as having potency against SARS-CoV-2 in vitro [6]. However, it is premature to speculate that sulfate polysaccharides may act to inhibit SARS-CoV-2. A novel glycosaminoglycan (GAG)-binding motif at the S1/S2 proteolytic cleavage site and two other GAG-binding-like motifs within SARS-CoV-2 spike glycoprotein (SGP) were found. Heparin sulfate oligosaccharide preferably interacts with GAG-binding motifs, which suggests a mechanism of SARS-CoV-2 binding to prevent invasion of a host cell [7]. However, whether all sulfate polysaccharides can inhibit SARS-CoV-2 entry to a host cell and what the structural characteristics depend on remain unknown.

A sulfated polysaccharide (AGSP) has been isolated from the gonad of *Haliotis discus hannai* Ino. The main structure of AGSP is →3)-GlcA(1→3)-Gal(1→ with sulfated branches consisting of prevalent Gal and minor Glc [8,9]. Its ability to enhance the osteoblast differentiation of C2C12 cells induced by BMP-2 has been confirmed [8].

In the present study, AGSP and deeply sulfate-modified AGSP (S-AGSP) were tested for their inhibitory activities against SARS-CoV-2 in vitro; as AGSP and S-AGSP share a primary molecular structure, comparison of their inhibitory activities against SARS-CoV-2 would reveal the essential structure property of sulfate polysaccharide against SARS-CoV-2. We also tested the interference effect of both polysaccharides on the interaction between spike glycoprotein and ACE2 using a biolayer interferometry (BLI) assay.

## 2. Materials and Methods

### 2.1. Materials

Abalone gonad sulfated polysaccharide (AGSP) was isolated from *Haliotis discus hannai* Ino as previously reported [8]. Standard MW dextran was purchased from Pharmacia Co. (Uppsala, Sweden). Ammonium acetate was purchased from Aladdin (Shanghai, China). All chemicals used in this study were analytical grade.

### 2.2. Experimental Methods

Infrared radiation (IR) spectroscopic measurement was carried out using a Specord 75 IR spectrometer (Carl Zeiss, Jena, Germany) on KBr pellets at room temperature. Nuclear magnetic resonance (NMR) spectroscopic analysis was recorded using a Bruker Ascend 400 spectrometer. Dried AGSP was dissolved in deuteroxide and lyophilized three times. The sample was then analyzed using NMR in a deuteroxide solution at 400 MHz (^1^H) and 100 MHz (^13^C).

### 2.3. Sulfation of AGSP

The sulfation of AGSP was conducted according to our previously reported method [10]. Briefly, anhydrous N, N-dimethylformamide (20 mL) and AGSP (1 g) were thoroughly mixed, and then a mixture of chlorosulfonic acid and pyridine with a volume ratio of 1:5 (25 mL) was added. The mixture was maintained at 60 °C for 3 h for sulfation reaction. After that, it was blended with 100 mL of ice water and adjusted to pH 7.0 with 20% NaOH to terminate the reaction. Three folds of 95% (*v*/*v*) ethanol were added to precipitate the sulfate product for 24 h at 4 °C. The precipitate was re-dissolved, dialyzed and lyophilized to yield a deeply sulfate-modified AGSP (S-AGSP). The sulfate content of polysaccharide was determined according to the BaCl_2_-gelatin method, using potassium sulfate as a standard [11,12]. In brief, AGSP or S-AGSP was hydrolyzed in 1.0 M HCl at 110 °C for 4 h. Next, 200 µL of the hydrolyzed sample was thoroughly mixed with 3% trichloroacetic acid (*v*/*v*) and BaCl_2_-gelatin solution. The mixture was placed at room temperature for 15 min, and then absorbance was measured at 360 nm using a microplate reader (Infinite M200, Tecan Infinite, Männedorf, Switzerland). IR spectrum analysis was used to identify the sulfation results.

### 2.4. Homogeneity and Molecular Weight (MW) Determination

The homogeneity and MW determination of AGSP and S-AGSP were conducted using a liquid chromatography instrument, Waters 2695 (Waters Co., Milford, MA, USA), equipped with a 7.8 × 300 mm TSK-gel G4000PWXL column (Tosoh Bioscience, Tokyo, Japan), and a 2414 refractive index detector (Waters Co., Milford, MA, USA), respectively. A sample solution of 20 µL (2 mg/mL) was injected into the system for each run. Ammonium acetate solution (0.1 mol/L) was used as eluent at a flow rate of 0.4 mL/min. The column was previously calibrated using standard dextrans, including 1000, 5000, 25,000, 50,000, 150,000, 410,000 and 670,000 Da.

### 2.5. Cell Culture and Cell Viability

A Vero E6 cell line was obtained from Procell Life Science & Technology Co., Ltd. (Wuhan, Shanghai). Cells were cultured using Dulbecco’s modified Eagle’s medium (DMEM; HyClone, Logan, UT, USA) containing 10% fetal bovine serum (FBS; Biochrom, Berlin, Germany) and 1% penicillin/streptomycin solution at 37 °C in a humidified atmosphere with 5% CO_2_. Vero E6 cells were precultured on 96-well plates at a density of 5 × 10^4^ cells/mL for 24 h. The medium was replaced and different concentrations of AGSP or S-AGSP (0, 0.4, 0.9, 1.8, 3.7, 7.5, 15, 30, 40 and 50 µg/mL) were added. After 24 h of incubation, 20 µL of 5 mg/mL 3-(4,5-dimethylthiazol-2-yl)-2,5-diphenyltetrazolium bromide (MTT) was added to each well for another 4 h of incubation. Next, MTT was replaced with 150 µL dimethyl sulfoxide (DMSO) and the plates were shaken for 8 min. The absorbance reading was recorded at 490 nm. All experiments were performed in triplicate.

### 2.6. Virus Neutralization Assay

The SARS-CoV-2 strain was isolated from COVID-19 patients in Shanghai, China. A SARS-CoV-2 neutralization assay was conducted in a biologically safe protected third-level laboratory at Naval Medical University. The assay process was performed as previously described by Song [6]. Sample concentrations tested were 30 µg/mL, 15 µg/mL, 7.5 µg/mL, 1.87 µg/mL, 0.94 µg/mL and 0.47 µg/mL. Immunofluorescent assays of the antibody against SARS-CoV-2 nucleocapsid protein showed SARS-CoV-2 as green and DAPI staining for the DNA of live Vero E6 cells showed Vero E6 cell nuclei as blue in immunofluorescent images. The relative intensity of SARS-CoV-2 was quantified using ImageJ analysis software (NIH, Bethesda, MA, USA). The ratio of green to blue in the images was quantified. Analyses compared results between experimental groups and the blank group.

### 2.7. Binding Affinity Measurements

A biolayer interferometry assay was used to characterize the interference of AGSP or S-AGSP in the binding interaction between SGP (Sino Biological Inc., Beijing, China) and ACE2 (Sino Biological Inc., Beijing, China). The assay was performed using an Octet Red 96 system (FortéBIO, Pall Forte BioCorp., Fremont, CA, USA) with Streptavidin biosensor tips (ForteBio, MenloPark, CA, USA). Briefly, 1 mg biotin was dissolved in 175 µL DMSO to obtain the biotin reaction solution. Next, 0.1 mg SGP was added to an 800 µL biotin reaction solution. The biotinylated SGP solution was obtained via desalination and centrifugation. The protocols were as follows. (1) Baseline subtraction was performed using SA biosensor tips (ForteBio, Menlo Park, CA, USA) dipped in PBS-Tween-20 buffer for the initial baseline for 2 min. (2) Biotinylated SGP loading for 10 min; sensors were dipped into wells containing PBS-BSA buffer for quenching for 10 min, followed by a 2 min incubation in PBS-Tween-20 solution to remove excess proteins and establish a new baseline. (3) Sensors were dipped into wells containing ACE2 or ACE2 with polysaccharide samples for 25 min as an association step. (4) Tips were dipped into PBS-Tween-20 buffer for 7 min for dissociation. The same procedure was followed using a PBS-Tween-20 buffer substitution for samples in a blank control.

### 2.8. Statistical Analysis

All experiments were performed in triplicate. One-way analysis of variance (ANOVA) was performed using SPSS 23.0 statistical software (SPSS Inc., Chicago, IL, USA). Data are presented as mean ± standard deviation (SD). Comparisons that yielded *p* < 0.05 were considered significant.

## 3. Results

### 3.1. Characteristics of AGSP and S-AGSP

**Figure 1:**
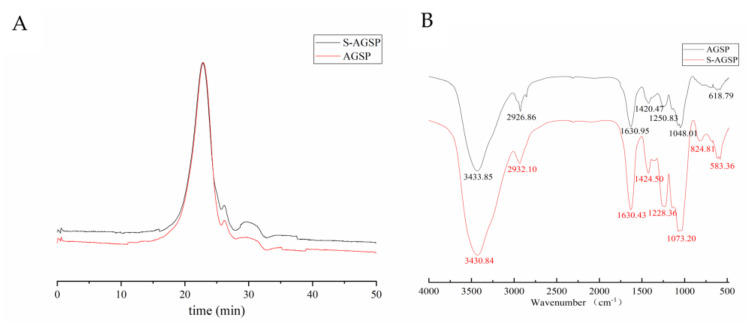
Characteristics of AGSP and S-AGSP. (**A**) Size exclusion chromatography profile of AGSP and S-AGSP. (**B**) IR spectra of AGSP and S-AGSP in the range of 4000–500 cm^–1^.

AGSP is a sulfated polysaccharide with an average MW of 11.85 kDa. It showed one symmetric peak in the size exclusion chromatography profile, which suggested that AGSP is homogeneous (Figure 1A). After the sulfuric acid reaction, the sulfate content increased from 6.82% (*w*/*w*) in AGSP to 11.87% (*w*/*w*) in S-AGSP and the reaction product remained a homogeneous fraction. The MW of S-AGSP was 13.72 kDa, which was increased slightly compared with that of AGSP.

### 3.2. IR Spectroscopic Analysis

AGSP and S-AGSP showed typical polysaccharide absorption peaks in the IR spectrum (Figure 1B). Compared with AGSP’s IR spectrum, a distinctive band boosting up at 1228.36 cm^−1^ in S-AGSP’s IR spectrum was observed, which was related to >S=O stretching [10]. The strong absorption of S-AGSP at 824.81 cm^−1^ illustrated that sulfate groups were equatorial [13]. These results suggested that S-AGSP contains more sulfate groups than AGSP, which agreed with the results of the sulfate content assay.

### 3.3. Sulfate Substituent Position Analysis Using NMR Spectra

**Figure 2:**
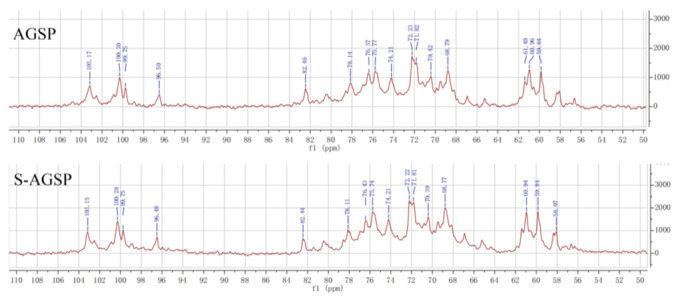
^13^C-NMR spectra of AGSP and S-AGSP, respectively.

NMR spectra of AGSP and S-AGSP were compared to identify the sulfate substituent position after the sulfated derivatization reaction. The ^1^H-NMR (Figure S1) and ^13^C-NMR (Figure 2) of AGSP and S-AGSP showed almost identical signal peaks, except for strengthening of the chemical shift at 75.74 ppm in the ^13^C-NMR spectra of S-AGSP. No new chemical shift signal was detected in the ^1^H-NMR spectra of S-AGSP compared with that of AGSP. This result confirmed the ^13^C-NMR spectra result, which indicated that the sulfate group position in S-AGSP was the same as that of AGSP. The sulfate position in AGSP was previously reported to be at C4 of →3)-α-Gal (1→ residue [8]. Thus, the sulfate position in S-AGSP was also confirmed to be substituted at C4 of →3)-α-Gal (1→ residue.

### 3.4. Cytotoxic Effects of AGSP and S-AGSP on Vero E6 Cells

**Figure 3:**
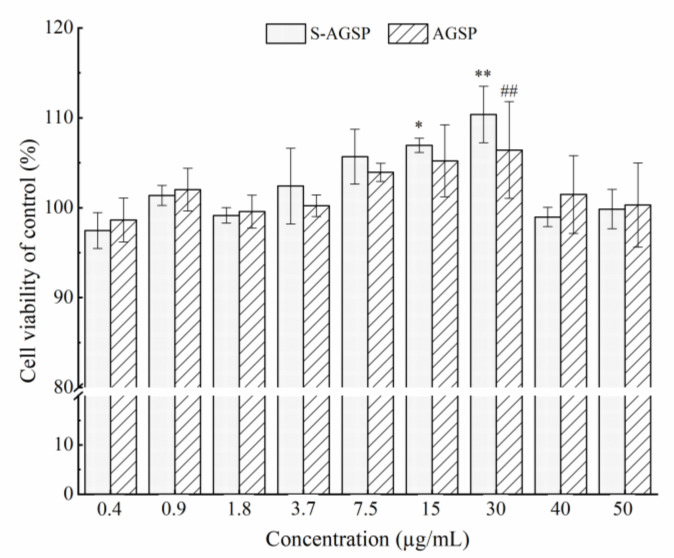
Effects of AGSP and S-AGSP on Vero E6 cell viability at concentrations ranging from 0 to 50 µg/mL. Data are presented as mean ± SD (*n* = 5). The ‘*’ indicates *p* < 0.05, ‘**’ and ‘^##^’ indicate *p* < 0.01.

AGSP and S-AGSP effects on Vero E6 cell viability were assessed using the MTT method to confirm safety. Neither AGSP nor S-AGSP affected the viability of Vero E6 cells in concentrations ranging from 0 to 50 µg/mL; on the contrary, AGSP and S-AGSP significantly promoted the proliferation of Vero E6 cells at 30 µg/mL (Figure 3).

### 3.5. Inhibitory Effects of AGSP and S-AGSP against SARS-CoV-2

**Figure 4:**
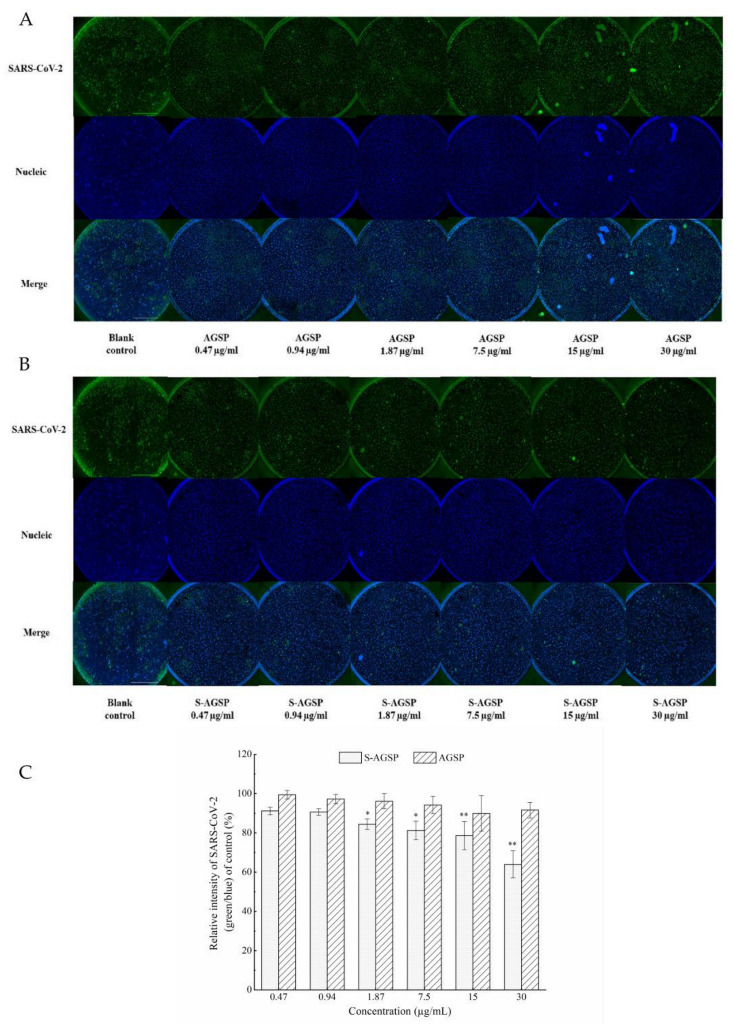
The effects of AGSP and S-AGSP against SARS-CoV-2. Representative images of immunofluorescent assays of (**A**) AGSP and (**B**) S-AGSP against SARS-CoV-2 for concentrations ranging from 0.47 to 30 µg/mL. (**C**) Quantitative calculation of the inhibition effects of AGSP and S-AGSP on SARS-CoV-2 (green/blue: SARS-CoV-2/live Vero E6 cells). Immunofluorescent assays of the antibody against SARS-CoV-2 nucleocapsid protein showed green and DAPI staining for the DNA of live Vero E6 cells showed blue. Data are presented as mean ± SD (*n* = 3). * *p* < 0.05, ** *p* < 0.01.

The inhibitory effects of AGSP and S-AGSP against SARS-CoV-2 infection in Vero E6 cells were tested. Representative images of the immunofluorescent assay showed the inhibition effects of AGSP and S-AGSP on SARS-CoV-2 infecting Vero E6 cells at various concentrations (Figure 4). The ratio of green to blue indicated the SARS-CoV-2 infection rate in Vero E6 cells. When the concentration of S-AGSP was increased, the ratio of virus to live cells declined (Figure 4B). This result indicated that S-AGSP inhibited the SARS-CoV-2 infection of Vero E6 cells. AGSP showed no significant difference compared with the blank control group for concentrations ranging from 0.47 to 30 µg/mL. However, S-AGSP showed obvious inhibition of SARS-CoV-2 infection in concentrations as low as 1.87 μg/mL (Figure 4C).

### 3.6. Inhibitory Effects of AGSP and S-AGSP on the Interaction between SGP and ACE2

**Figure 5:**
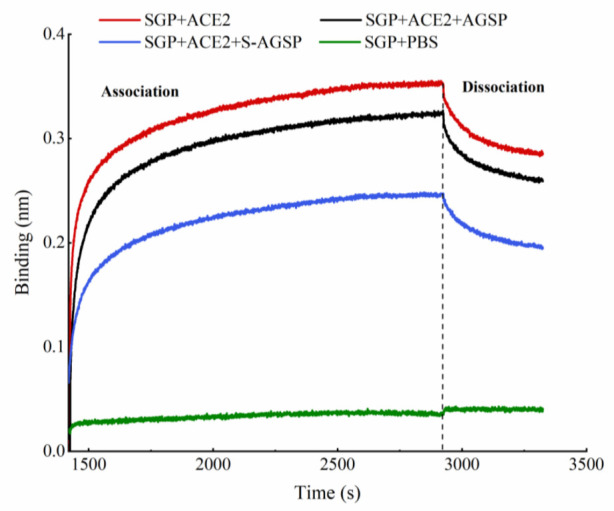
Biolayer interferometry affinity measurements of SGP to ACE2 in the presence of AGSP or S-AGSP. Red line: SGP with ACE2; black line: SGP with ACE2 and AGSP; blue line: SGP with ACE2 and S-AGSP; green line: SGP with PBS. Association–dissociation curves illustrate dissimilar affinities when AGSP or S-AGSP interfered.

The inhibitory effects of AGSP and S-AGSP on the interaction between SGP and ACE2 were determined using BLI. First, SGP formed a stable layer on the surfaces of SA sensor tips. SGP-coated SA sensors were then incubated with ACE2 in the presence of AGSP or S-AGSP to measure corresponding association and dissociation signals. The interference intensities of AGSP or S-AGSP on binding affinity were observed. No valid signal was observed in the blank control group, whereas association and dissociation signals were observed for SGP to ACE2. These results indicated effective binding between SGP and ACE2, as evident from the SA sensor (Figure 5). The association–dissociation curves showed that SGP still had a high adsorption binding affinity to ACE2 in the presence of AGSP. However, when S-AGSP was added to the incubated mixture, the adsorption affinity of SGP to ACE2 decreased. S-AGSP significantly weakened the binding affinity between these two proteins. The BLI experiment revealed that the sulfate content of the polysaccharide played a critical role in influencing the binding affinity of SGP to ACE2.

## 4. Discussion

The original AGSP polysaccharide was a sulfated polysaccharide. The sulfate-modified product S-AGSP had the same ^1^H-NMR and ^13^C-NMR spectra signals as the AGSP. These results indicated that the sulfate groups added to the AGSP residues during sulfate modification were in the same position as in the original AGSP. A previous study revealed that the sulfate group in the AGSP is at C4 of →3)-α-Gal (1→ residue [8]. Thus, the sulfate group in the S-AGSP was also at C4 of →3)-α-Gal (1→ residue. Generally, the specificity of sulfation during modification cannot be controlled; it depends on the intrinsic molecular structure of the original polysaccharide. Hence, the judgement that the sulfate group in S-AGSP substituted in the same AGSP residue position is reasonable.

Recent findings showed that GAG-binding-like motifs located at the SARS-CoV-2 spike glycoprotein (SGP) and the binding of SGP to the ACE2 host receptor facilitated viral entry to host cells [7]. Based on this finding, it has been suggested that sulfated polysaccharides, such as heparin and some seaweeds’ polysaccharides, may be potential solutions for the global COVID-19 health problem [14,15]. In this study, the inhibition activities of AGSP and S-AGSP on SARS-CoV-2 infection of Vero E6 cells were tested. S-AGSP showed significant inhibition of SARS-CoV-2 infection of Vero E6 cells, yet no apparent inhibitory effect was observed for AGSP. AGSP and S-AGSP share the same monosaccharide compositions, branch types and sulfate substitution positions. Therefore, the inhibition of SARS-CoV-2 infection by S-AGSP ought to ascribe to the high sulfate level in S-AGSP, which reached 11.87%. The sulfate content of AGSP was only 6.82%. These results suggested that the sulfate content in polysaccharide was positively associated with the inhibition of SARS-CoV-2 infection.

SARS-CoV-2 utilizes a glycosylated spike protein (comprising an S1 subunit and an S2 subunit in each spike monomer) to bind to the host ACE2 [2,3]. Binding triggers a cascade of events that fuses host cell and viral membranes allowing the virus to enter the host cell [4]. SGP binding to the ACE2 receptor is a critical initial step for SARS-CoV-2 entry into target cells. We tested the interference of AGSP or S-AGSP in the SGP and ACE2 interaction using the BLI method. Results showed that S-AGSP strongly hampered binding between SGP and ACE2, compared with AGSP’s weak hindering effect. This result indicated that S-AGSP’s interference in SARS-CoV-2 infection of Vero E6 cells was facilitated by inhibiting the virus from binding to host cellular receptors. Our investigation found a novel insertion of glycosaminoglycan (GAG)-binding motif at the S1/S2 proteolytic cleavage site (681–686 (PRRARS)) and two other GAG-binding-like motifs within SARS-CoV-2 spike glycoprotein (SGP). An unbiased computational ligand docking showed that heparan sulfate could interact with the GAG-binding motif at the S1/S2 site when the receptor–binding domain was in an open conformation [7]. S-AGSP is a heavily sulfated polysaccharide with a strong negative charge. S-AGSP’s inhibition of SGP’s binding to ACE2 receptors may be due to S-AGSP’s docking with the GAG-binding motif at the S1/S2 site. In contrast, AGSP possesses less sulfate, which results in less interaction with the GAG-binding motif.

## 5. Conclusions

AGSP and S-AGSP hold identical molecular structures with different sulfate contents. S-AGSP showed an obvious inhibitory effect on SARS-CoV-2 in vitro, whereas AGSP did not. S-AGSP significantly weakened the interaction between SARS-CoV-2 spike glycoprotein and ACE2. The polysaccharide’s sulfate content played an important role in negatively influencing the binding affinity of SGP to ACE2.

## Data Availability

All data are contained within the article.

## Supplementary Materials

The following are available online at https://www.mdpi.com/article/10.3390/foods11182865/s1, Figure S1: ^1^H-NMR spectra of AGSP and S-AGSP.
