# Supplementary figures and images for: A Sulfated Abalone Polysaccharide Inhibited SARS-CoV-2 Infection of Vero E6 Cells In Vitro
Source: Foods. 2022 Sep 16;11(18):2865. doi: 10.3390/foods11182865 (PMC9498428; doi:10.3390/foods11182865)

## Supplementary material

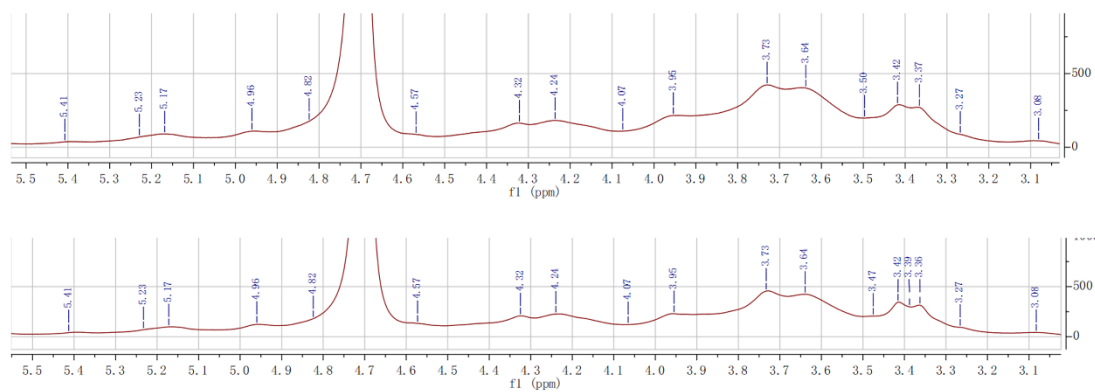

**Figure S1.**  $^1\text{H}$ -NMR spectra of AGSP and S-AGSP.

Supplement: Supplementary file 1 [file foods-11-02865-s001.zip › foods-1830233-supplementary.pdf]
